# Recent Advances in Visible Light-Induced C-H Functionalization of Imidazo[1,2-a]pyridines

**DOI:** 10.3390/molecules30030607

**Published:** 2025-01-30

**Authors:** Juanjuan Gao, Xinlei Fu, Kai Yang, Zhaowen Liu

**Affiliations:** Jiangxi Province Key Laboratory of Pharmacology of Traditional Chinese Medicine, College of Pharmacy, Gannan Medical University, Ganzhou 341000, China; gaoya0758@163.com (J.G.); fxl05020502@163.com (X.F.); kai_yang@gmu.edu.cn (K.Y.)

**Keywords:** imidazo[1,2-a]pyridine, C-H functionalization, visible light-induced, synthesis

## Abstract

The imidazo[1,2-a]pyridine skeleton is widely present in many natural products and pharmaceutical agents. Due to its impressive and significant biological activities, such as analgesic, anti-tumor, antiosteoporosis, and anxiolytic properties, the derivatization of imidazo[1,2-a]pyridine skeleton has attracted widespread attention from chemists. In recent years, significant progress has been made in the derivatization of imidazo[1,2-a]pyridines through direct C-H functionalization, especially through visible light induction. This review highlights recent advances in visible light-induced C-H functionalization of imidazo[1,2-a]pyridines during the past ten years, and some reaction mechanisms are also discussed.

## 1. Introduction

**Figure 1:**
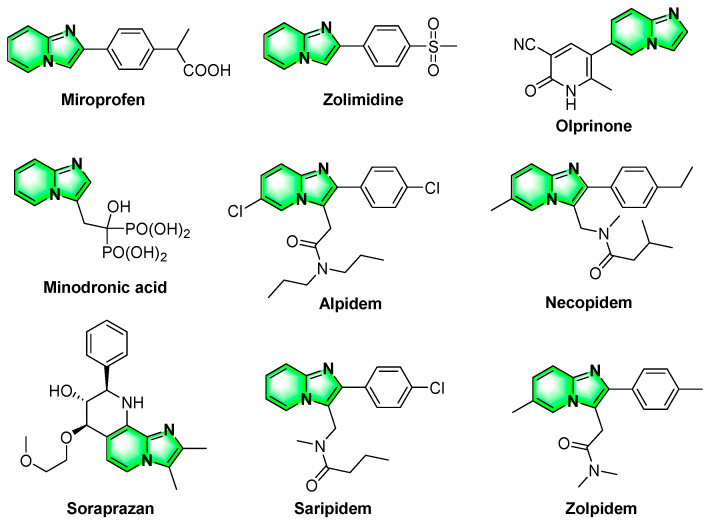
Examples of marketed drugs containing imidazo[1,2-a]pyridine skeleton.

Imidazo[1,2-a]pyridine is an important class of nitrogen-containing heterocyclic compounds and widely presents in natural products, pharmaceutical molecules, functional materials, and agrochemicals [1,2]. Imidazo[1,2-a]pyridine derivatives have important biological activities and pharmacological effects, including anticancer [3], antituberculosis [4], antibacterial [5], antiviral [6], anti-inflammatory [7], antidiabetic [8], and antiplasmodial [9] properties, etc. Moreover, some marketed drugs for clinical use, such as Miroprofen, Zolimidine, Olprinone, Minodronic acid, Alpidem, Necopidem, Soraprazan, Saripidem, and Zolpidem, are derived from the skeleton of imidazo[1,2-a]pyridine (Figure 1). Therefore, the development of the functionalization methods of imidazo[1,2-a]pyridine derivatives has important application value and practical significance [10,11].

So far, a series of synthetic methods have been developed to obtain diverse imidazo[1,2-a]pyridines. The strategy of C-H bond functionalization is considered a powerful approach for rapidly introducing functional groups into imidazo[1,2-a]pyridine skeleton, due to its relative straightforwardness, high atomic economy, and synthetic step economy [12,13,14,15]. However, this strategy still has some limitations, including harsh reaction conditions, strong oxidants, and expensive metal salt catalysts [16,17,18,19]. There is still a lot of room for developing green, simple, and sustainable synthesis methods. Photochemical synthesis is considered one of the most energy-efficient and eco-friendly methods, and can promote the activation of organic molecules through single electron transfer (SET) processes, thereby achieving various organic transformations [20,21,22,23]. In recent years, more and more chemical workers have achieved C-H functionalization of imidazo[1,2-a]pyridines through photochemical synthesis strategies. This article provides an overview of the relevant research in this field over the past ten years, with the aim of providing assistance for future research in this area.

## 2. C-C Bond Formation

### 2.1. C3-Fluoroalkylation of Imidazo[1,2-a]pyridines

**Scheme 1:**
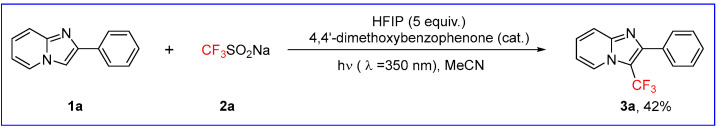
Trifluoromethylation of 2-phenylimidazo[1,2-a]pyridine with sodium triflinate.

Fluoroalkyl fragments play a crucial role in the fields of pharmaceuticals and materials science. The introduction of fluoroalkyl groups into heterocyclic frameworks can cause significant changes in the physicochemical and biological properties of the compounds [24,25]. Therefore, great attention has been paid to developing efficient synthesis methods for constructing fluoroalkyl imidazo[1,2-a]pyridine derivatives [26,27,28]. Langlois’ reagent (sodium triflinate, CF_3_SO_2_Na), a classic trifluoromethylation reagent, is commonly used to incorporate the CF_3_ group into imidazo[1,2-a]pyridine frameworks [29]. In 2016, Rueping’s study group first reported the trifluoromethylation of imidazo[1,2-a]pyridines under near-UV irradiation (λ = 350 nm) by using 4,4′-dimethoxybenzophenone as a photoredox catalyst (Scheme 1) [30]. However, this article only reported an example of the C3-trifluoromethylation of imidazo[1,2-a]pyridine, providing 2-phenyl-3-trifluoromethylimidazo[1,2-a]pyridine **3a** in a 42% yield.

**Scheme 2:**
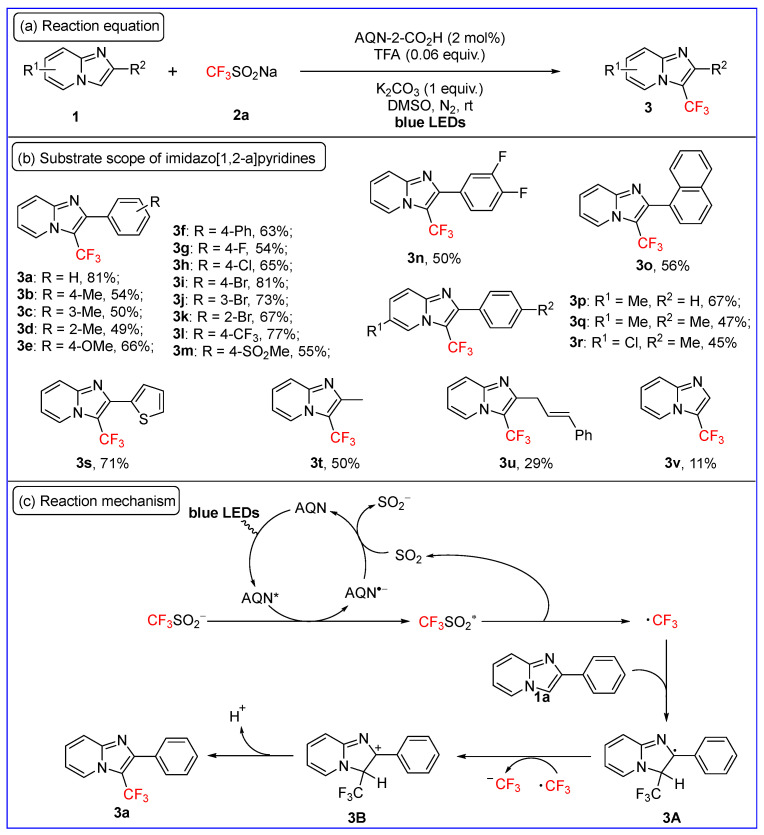
Visible light-mediated trifluoromethylation of imidazo[1,2-a]pyridines with sodium triflinate. (**a**) Reaction equation. (**b**) Substrate scope of imidazo[1,2-a]pyridines. (**c**) The plausible reaction mechanism.

In 2019, Zhang’s study group [31] reported a transition-metal-free, visible light-induced method for the trifluoromethylation of imidazo[1,2-a]pyridines, by using anthraquinone-2-carboxylic acid (AQN-2-CO_2_H) as a photoorganocatalyst in the presence of K_2_CO_3_ and trifluoroacetic acid (Scheme 2a). Under the optimized reaction conditions, the authors subsequently investigated the substrates’ scope (Scheme 2b). A variety of substituents, such as methyl, methoxyl, phenyl, fluoro, chloro, bromo, trifluoromethyl, and methylsulfonyl groups on the C2-phenyl ring of 2-phenyl imidazo[1,2-a]pyridines were well tolerated under the standard conditions, giving the desired products **3a**–**3m** in 49–81% yields. Moreover, 2-(3,4-Difluorophenyl)imidazo[1,2-a]pyridine and 2-(naphthalen-1-yl)- imidazo[1,2-a]pyridine were also suitable for this conversion, to afford the corresponding products **3n** and **3o**, respectively. Substrates with the methyl or halogen group on the pyridine ring could proceed smoothly under the standard conditions and provided the desired products **3p**–**3r**. Both 2-thienylimidazo[1,2-a]pyridine and 2-methylimidazo- [1,2-a]pyridine could also provide the corresponding products **3s** and **3t** in 71% and 50% yields, respectively. In addition, 2-Alkenyl chain imidazo[1,2-a]pyridine and imidazo[1,2-a]pyridine could be converted into the desired products under the standard conditions, but the yields were relatively low (**3u**, 29% and **3v**, 11%).

The radical pathway of the reaction was established by radical scavenging experiments. The plausible mechanism of this reaction has been described in Scheme 2c. Firstly, CF_3_ radical was generated through photoredox-catalyzed cyclization reactions. Subsequently, the CF_3_ radical reacted with imidazo[1,2-a]pyridine to form the radical intermediate **3A**. Then, **3A** was oxidized by the CF_3_ radical to obtain the carbocation intermediate **3B**. Finally, **3B** underwent deprotonation to form the final product **3**.

**Scheme 3:**
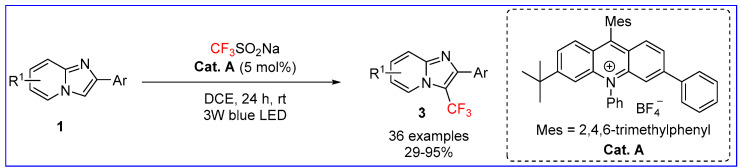
Visible light-promoted trifluoromethylation of 2-arylimidazo[1,2-a]pyridines using mesityl acridinium as a photoredox catalyst.

In 2019, Cui’s study group also achieved visible light-promoted C-H trifluoromethylation of imidazopyridines by using mesityl acridinium as a photoredox catalyst at room temperature (Scheme 3) [32]. Unfortunately, the unsubstituted imidazo[1,2-a]pyridines on the C2 position could not proceed smoothly in this catalytic system.

**Scheme 4:**
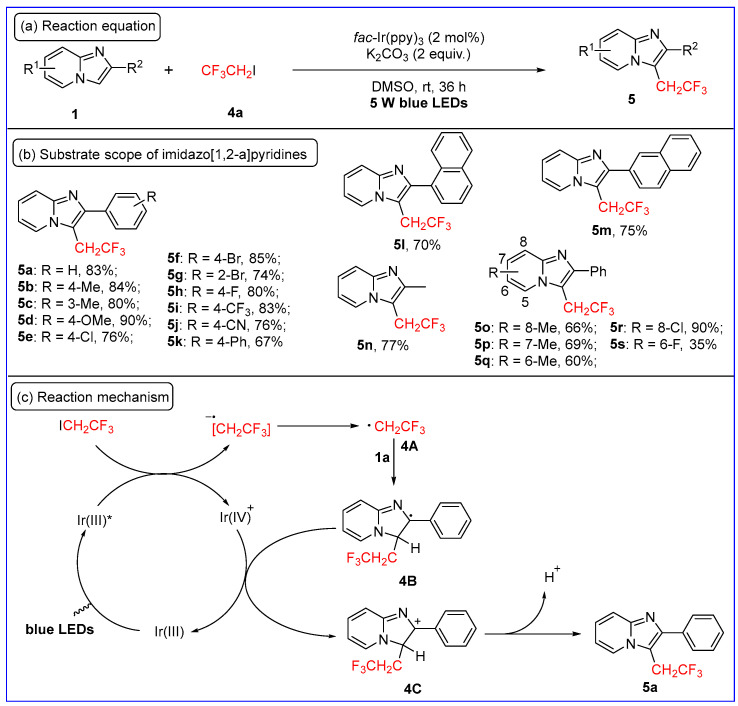
Visible light-promoted trifluoroethylation of imidazo[1,2-a]pyridines with 1,1,1-trifluoro-2-iodoethane. (**a**) Reaction equation. (**b**) Substrate scope of imidazo[1,2-a]pyridines. (**c**) The plausible reaction mechanism.

Polyfluoroalkyl halides can also be used for the visible light-induced polyfluoroalkylation of imidazo[1,2-a]pyridines. For example, Xu’s research group developed a visible light-catalyzed C3 trifluoroethylation of imidazo[1,2-a]pyridines by using 1,1,1-trifluoro-2-iodoethane **4a** as a trifluoroethyl reagent (Scheme 4a) [33]. The photocatalyst *fac*-Ir(ppy)_3_ was essential for this conversion. Various substituted 2-arylimidazopyridines could obtain the corresponding products with moderate to excellent yields (Scheme 4b), indicating that the protocol has good substrate applicability. It is noteworthy that 2-methylimidazo[1,2-a]pyridine was also suitable for this conversion, yielding the desired product **5n** in a 77% yield.

A plausible mechanism was presented in Scheme 4c. Initially, *fac*-Ir(ppy)_3_ was transformed into an excited state [*fac*-Ir(III)(ppy)_3_*] under light irradiation, and then oxidized by CF_3_CH_2_I to generate a [*fac*-Ir(IV)(ppy)_3_]^+^ complex and a •CH_2_CF_3_ radical species **4A**. Subsequently, **4A** reacted with imidazo[1,2-a]pyridine **1a** to form the radical intermediate **4B**, and was oxidized by [*fac*-Ir(IV)(ppy)_3_]^+^ to produce the carbocation intermediate **4C**. Finally, **4C** underwent deprotonation to form the final product **5a**.

**Scheme 5:**
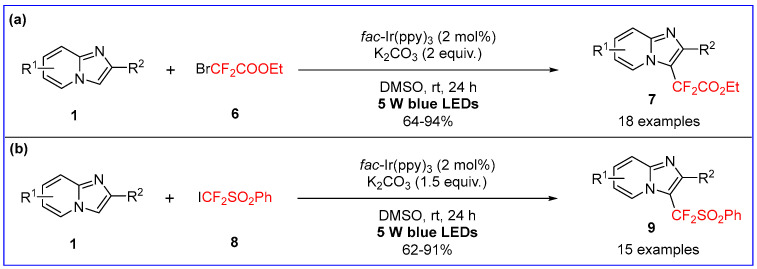
(**a**) Visible light-induced photocatalytic difluoroacetylation of imidazo[1,2-a]pyridines. (**b**) Visible light-induced (phenylsulfonyl)difluoromethylation of imidazo[1,2-a]pyridines.

A similar synthetic strategy can also achieve the difluoroacetylation and (phenylsulfonyl)difluoromethylation of imidazo[1,2-a]pyridines, respectively [34,35]. For example, Fu’s research group developed a visible light-mediated protocol for direct difluoroacetylation of imidazo[1,2-a]pyridines with BrCF_2_CO_2_Et by using *fac*-Ir(ppy)_3_ as a photocatalyst in the presence of K_2_CO_3_, yielding the corresponding products in 64–94% yields (Scheme 5a) [34]. PhSO_2_CF_2_I also achieved the same transformation, resulting in the (phenylsulfonyl)difluoromethylation of imidazo[1,2-a]pyridines in 62–91% yields (Scheme 5b) [35].

**Scheme 6:**
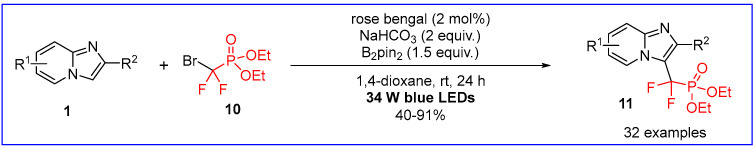
Visible light-induced difluoromethylenephosphonation of imidazo[1,2-a]pyridines.

In addition to metal photocatalysts, many organic photocatalysts also exhibit good catalytic activity in visible light-induced reactions, and are widely used in the field of organic synthesis. In 2019, Hajra’s research group reported the organophotoredox-catalyzed difluoromethylenephosphonation of imidazo[1,2-a]pyridines under visible light irradiation by using rose bengal (RB) as a photoredox catalyst (Scheme 6) [36]. The desired coupling product was not formed in the absence of Bis(pinacolato)diboron (B_2_pin_2_), indicating that its presence was essential in this reaction. Other heterocycles, such as imidazo[2,1-b]thiazole, benzo[d]imidazo-[2,1-b]thiazole, and indole, were also suitable for this conversion. Mechanism experiments showed that the reaction possibly proceeds through a single electron transfer (SET) process.

**Scheme 7:**
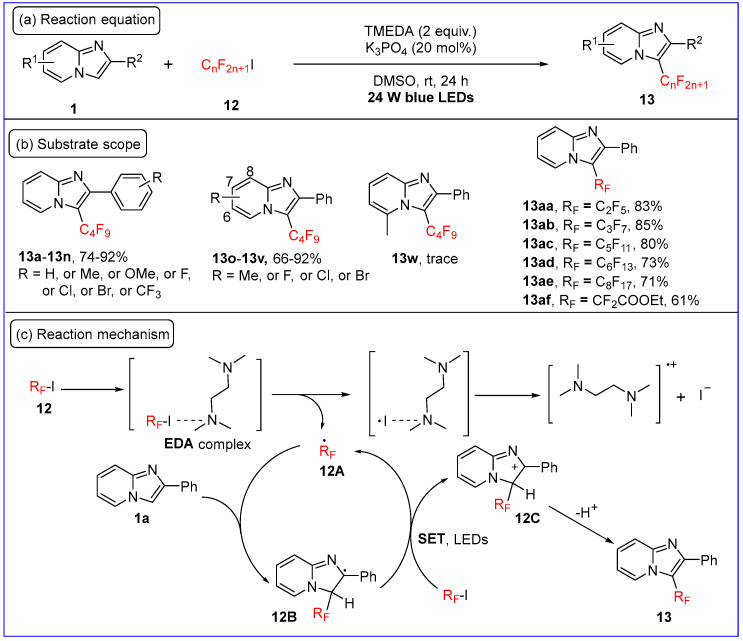
Visible light-induced perfluoroalkylation of imidazo[1,2-a]pyridines with perfluoroalkyl iodides. (**a**) Reaction equation. (**b**) Substrate scope. (**c**) The plausible reaction mechanism.

Photoinduced radical reactions driven by electron donor–acceptor (EDA) complexes are also widely used in the C-H functionalization of imidazo[1,2-a]pyridines. In 2021, Zhu’s group reported a visible light-induced perfluoroalkylation of the imidazo[1,2-a]pyridines with perfluoroalkyl iodides via photoactive EDA complexes at room temperature (Scheme 7a) [37]. This method showcases a broad substrate scope and wide functional group tolerance. A series of 3-perfluoroalkyl substituted imidazo[1,2-a]pyridines could be obtained with moderate to excellent yields (Scheme 7b). It should be noted that 5-methyl-2-phenylimidazo[1,2-a]pyridine could not provide the desired product **13w** under the conditions. A possible visible light-induced mechanism through photoactive EDA complexes was proposed in Scheme 7c [38,39]. In the same year, a similar strategy of perfluoroalkylation via photoactive EDA complexes and DUB as a base was reported by Wu’s group [40].

**Scheme 8:**
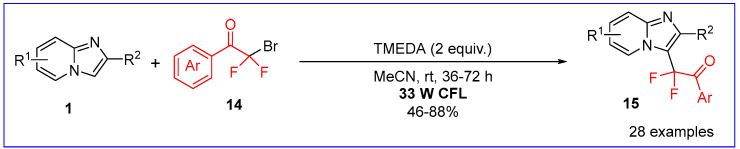
Visible light-induced difluoroalkylation of imidazo[1,2-a]pyridines with bromodifluoroaryl ketones.

In 2019, Xu et al. reported photochemical direct C-H difluoroalkylation of imidazo[1,2-a]pyridines with bromodifluoromethylaryl ketones via visible light irradiation (Scheme 8) [41]. Tetramethylethylenediamine (TMEDA), a mild organic base, played a key role in this reaction by removing the proton of a radical intermediate to obtain a radical anion. Compared with the 33 W compact fluorescent light (CFL), the reaction efficiency was very low under the 5 W blue light-emitting diode (LED), with a wavelength of only ~460 nm.

### 2.2. C3-Cyanomethylation of Imidazo[1,2-a]pyridines

**Scheme 9:**
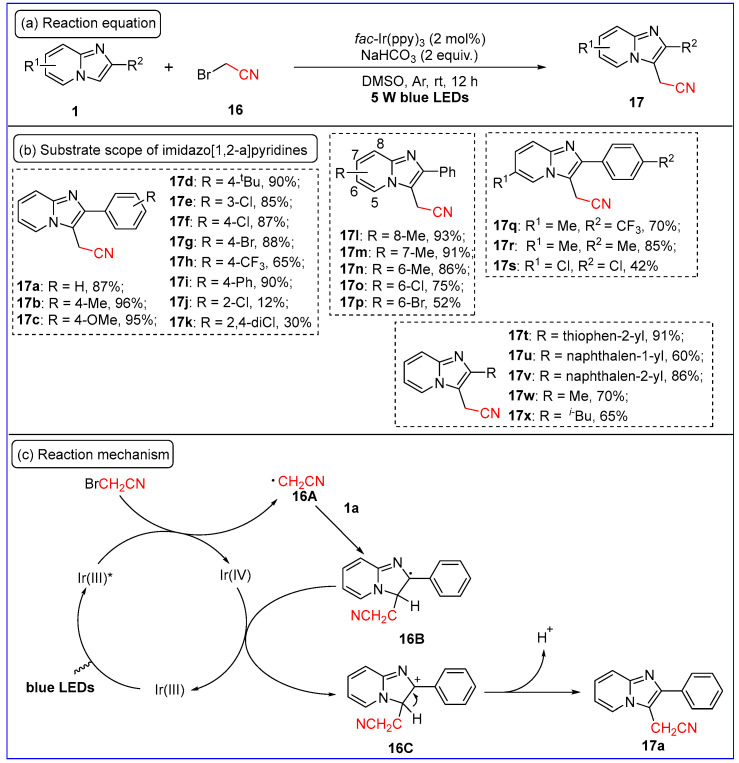
Visible light-induced C3-cyanomethylation of imidazo[1,2-a]pyridines with bromoacetonitrile. (**a**) Reaction equation. (**b**) Substrate scope of imidazo[1,2-a]pyridines. (**c**) The plausible reaction mechanism.

In the field of organic synthesis and drug synthesis, cyano groups can be converted into various useful functional groups [42]. Therefore, the cyanation of heterocyclic compounds has received widespread attention from organic chemists [43]. In 2017, Sun’s group explored a visible light-promoted protocol for C3 cyanomethylation of imidazo[1,2-a]pyridines with bromoacetonitrile by using *fac*-Ir(ppy)_3_ as a photocatalyst (Scheme 9a) [44]_._ The substrates bearing electron-donating groups (such as methyl, methoxyl, tertbutyl, and phenyl) and electron withdrawing groups (such as Cl-, Br-, and CF_3_) on the *para*- or *meta*- position of the benzene ring could produce the desired products **17a**–**17i** in 65–96% yields (Scheme 9b). However, the reaction efficiency of ortho-Cl substrates was significantly reduced (**17j**, 12% and **17k**, 30%). Furthermore, 2-Phenyl imidazo[1,2-a]pyridines with the Me-, Cl-, and Br- groups on the pyridine ring proceeded smoothly and provided the corresponding products **17l**–**17p** in moderate to excellent yields (52–93%). Imidazo[1,2-a]pyridines with an aryl group (such as thiophen-2-yl, naphthalen-1-yl, or naphthalen-2-yl) or an alkyl group (such as methyl or isobutyl) on the C2 position also worked well and afforded the corresponding products **17t**–**17x** in satisfactory yields. A possible mechanism for visible light-induced cyanomethylation by using *fac*-Ir(ppy)_3_ as a photocatalyst was proposed in Scheme 9c.

**Scheme 10:**
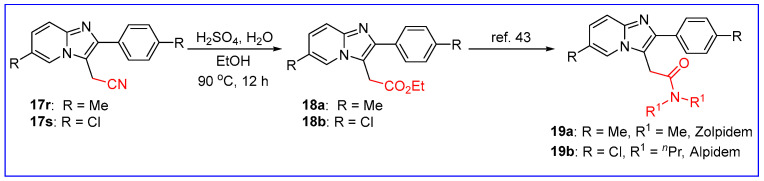
Synthesis of Zolpidem and Alpidem.

The effective synthesis of Zolpidem and Alpidem can be achieved via the derivatization of C3 cyanomethylation products [45]. Firstly, the 3-cyanomethylation products **17r** and **17s** undergo hydrolysis and an esterification reaction to obtain the corresponding esters **18a** and **18b**, followed by aminating to obtain Zolpidem (**19a**) and Alpidem (**19b**), respectively (Scheme 10).

### 2.3. C3-Aminoalkylation of Imidazo[1,2-a]pyridines

**Scheme 11:**
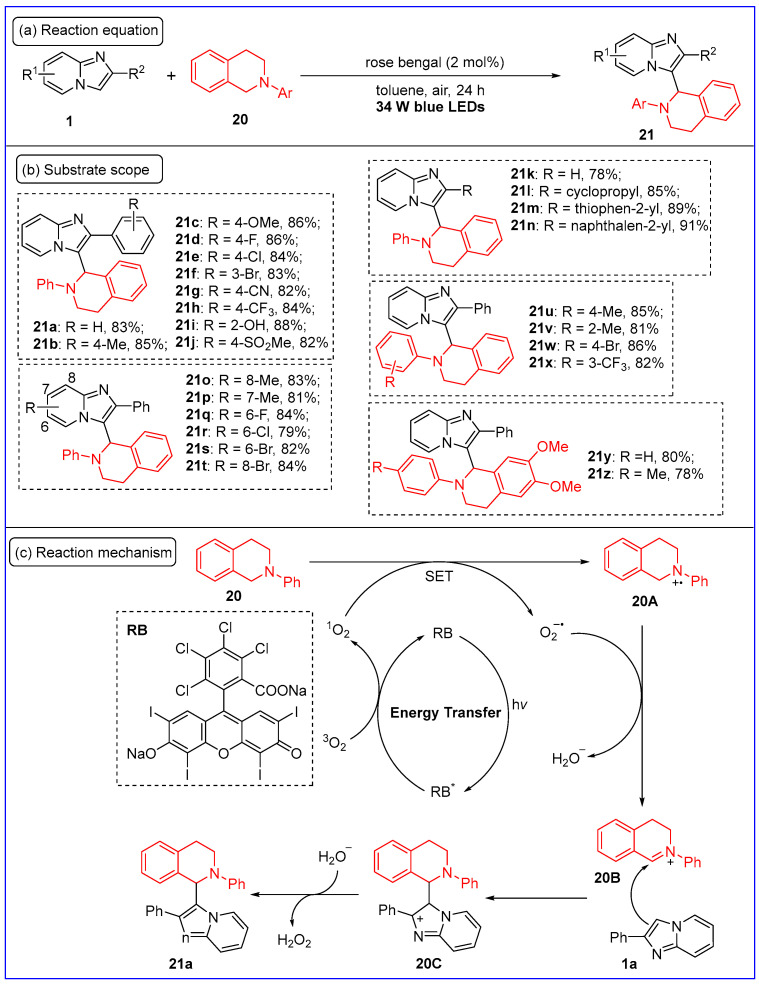
Visible light-promoted cross-dehydrogenative coupling of imidazo[1,2-a]pyridines with tertiary amine. (**a**) Reaction equation. (**b**) Substrate scope. (**c**) The plausible reaction mechanism.

The direct α-amino C-H functionalization by photocatalysis can achieve the preparation of important pharmaceutical skeletons via the formation of C-C and C-heteroatom bonds, which is of great significance in organic synthesis. In 2018, Hajra’s group reported an aerobic visible light-promoted oxidative cross-dehydrogenative coupling between imidazo[1,2a]pyridines and *N*-phenyltetrahydroisoquinoline by using rose bengal (RB) as a photocatalyst (Scheme 11a) [46].

Imidazo[1,2-a]pyridine substrates with a series of substituents, such as methyl, methoxyl, fluoro, chloro, bromo, cyan, trifluoromethyl, hydroxyl, methylsulfonyl, cyclopropyl, thiophen-2-yl, and naphthalen-2-yl on the benzene or pyridine ring were well tolerated, and gave the corresponding products **21a**–**21t** in 78–91% yields (Scheme 11b). Various substituted N-aryl tetrahydroisoquinolines bearing methyl, bromo, and trifluoromethyl groups could also provide the desired products **21u**–**21z** in 78–86% yields. A plausible mechanistic pathway was shown in Scheme 11c. Based on the redox potential values of excited state RB* and *N*-phenyltetrahydroisoquinoline, it is believed that this reaction may be involved in energy transfer processes rather than photoredox catalysis.

**Scheme 12:**
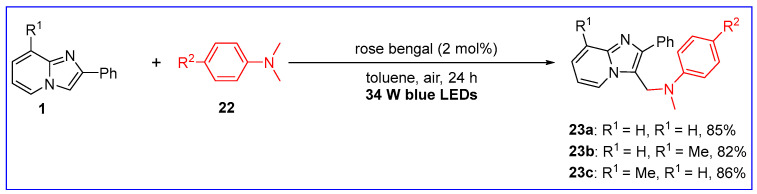
Visible light-induced aminoalkylation of imidazo[1,2-a]pyridines with N, N-dimethylaniline.

Excitingly, *N, N*-dimethylaniline substrates were also suitable for the protocol, giving the coupling aminomethylation products **23a**–**23c** in 82–86% yields (Scheme 12). However, only three examples were reported in this paper.

**Scheme 13:**
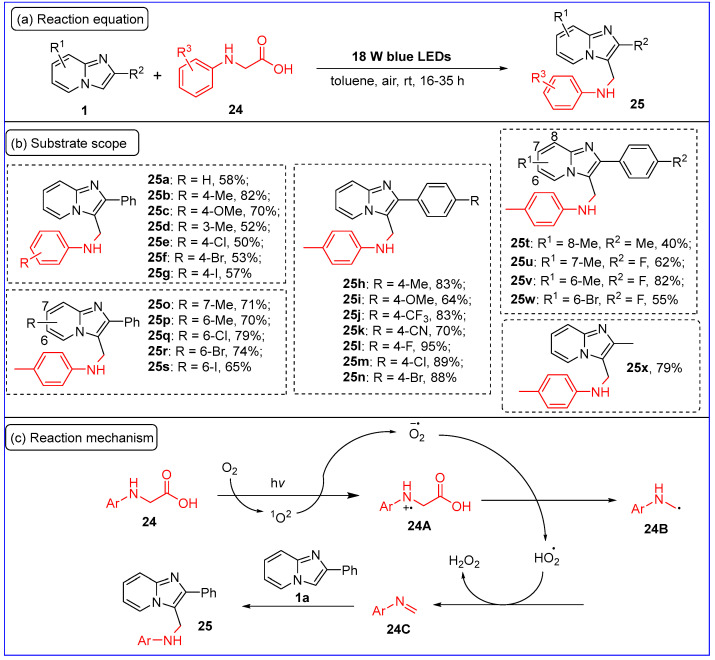
Visible light-promoted decarboxylative aminoalkylation of imidazo[1,2-a]pyridines with *N*-aryl glycines. (**a**) Reaction equation. (**b**) Substrate scope. (**c**) The plausible reaction mechanism.

Visible light-induced decarboxylation coupling is another strategy for achieving the aminomethylation of imidazo[1,2-a]pyridines [47]. In 2019, Le et al. reported visible light-promoted decarboxylative aminomethylation of imidazo[1,2-a]pyridines with N-aryl glycines in the air in the absence of expensive photosensitizers and extra additives at room temperature (Scheme 13a) [48]. As shown in Scheme 13b, an array of *N*-aryl glycines with electron-donating groups (such as methyl and methoxyl) or halogen (such as chloro, bromo, and iodine) on the aryl rings could react smoothly with 2-phenylimidazo[1,2-a]pyridines, providing the corresponding products **25a**–**25g** in 50–82% yields. Various imidazo[1,2-a]pyridines containing aryl and methyl substituents on the C2 position could also react with *N*-4-methylphenylglycine, providing the corresponding products **25h**–**25x** in 40–95% yields. Overall, this protocol exhibited good functional group tolerance and substrate applicability.

A plausible mechanism for this decarboxylative coupling process was also proposed, as shown in Scheme 13c. Firstly, singlet oxygen generated from O_2_ under blue LED illumination reacted with *N*-aryl glycine **24** to form amino cation radical **24A** and superoxide radical anion (O_2_^−•^). Subsequently, superoxide radical anion (O_2_ ^−•^) induced **24A** to undergo a proton transfer and decarboxylation process, transforming it into α-amino radical **24B**. Then, α-amino radical **24B** was further oxidized by HO_2_• to obtain imine intermediate **24C**. Finally, **24C** underwent electrophilic addition with 2-phenylimidazo[1,2-a]pyridine **1a** to obtain the final product **25**.

**Scheme 14:**
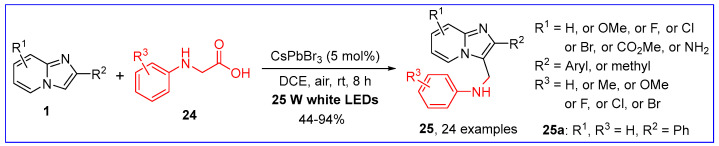
Visible light-induced aminoalkylation of imidazo[1,2-a]pyridines with *N*-aryl glycines.

In 2020, Yu et al. developed a decarboxylative coupling method for the direct aminomethylation of imidazo[1,2-a]pyridines with *N*-aryl glycines by using 5 mol% CsPbBr_3_ as a photocatalyst under the irradiation of white LEDs (Scheme 14) [49]. Both imidazo[1,2-a]pyridines and *N*-aryl glycine substrates with different substituents were investigated, providing the corresponding product **25** in 44–94% yields. The recycling experiments showed that the photocatalyst CsPbBr3 has a high catalyst economy, can be reused at least five times, and has no significant decrease in activity. Meanwhile, a gram-scale experiment (5 mmol) was also successfully carried out, obtaining the desired *N*-((2-phenylimidazo[1,2-a]pyridin-3-yl)methyl)aniline **25a** (1.32 g) in an 87% yield.

**Scheme 15:**
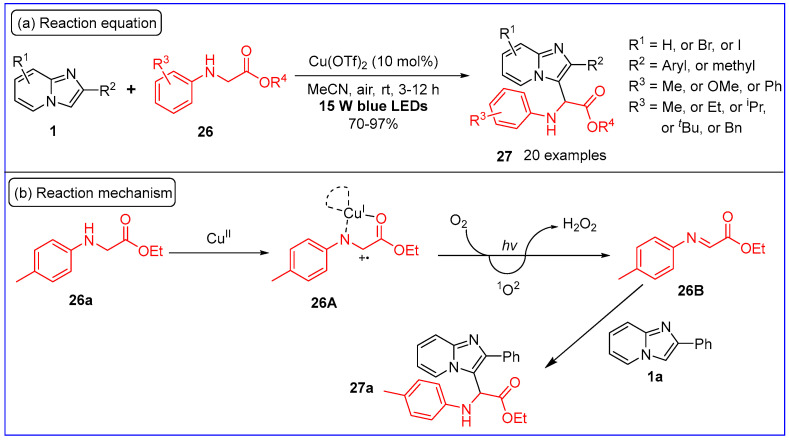
Visible light-induced cross-dehydrogenative coupling reaction of imidazo[1,2-a]pyridines with *N*-arylglycine esters. (**a**) Reaction equation. (**b**) The plausible reaction mechanism.

A dehydrogenation coupling reaction can also be achieved between imidazo[1,2-a]pyridines and *N*-arylglycine esters (**26**) under blue light irradiation by using copper (II) salt as the sole catalyst, resulting in α-heteroaryl-substituted α-amino acid ester derivatives (**27**) with good to excellent yields (Scheme 15a) [50]. It should be pointed out that *N*-arylglycine ester substrates with strong electron-withdrawing groups (cyano, nitro, and triflouromethyl) on the *N*-benzene rings could not transform to the desired products under the optimized conditions. The authors proposed a possible mechanism for this conversion, as shown in Scheme 15b. Initially, Copper (II) induced the conversion of *N*-aryl glycine ester **26a** into intermediate **26A** ([Cu(I)-NH^+•^]) via a single electron transfer (SET) [51]. Concurrently, singlet oxygen produced via the reaction of **26A** and molecular oxygen under visible light irradiation further oxidized the NH^+•^ of **26** into imine intermediate **26B**. Finally, the electrophilic addition process occurred between **26B** and **1a**, resulting in the desired product **27a**.

### 2.4. C3-Formylation of Imidazo[1,2-a]pyridines

**Scheme 16:**
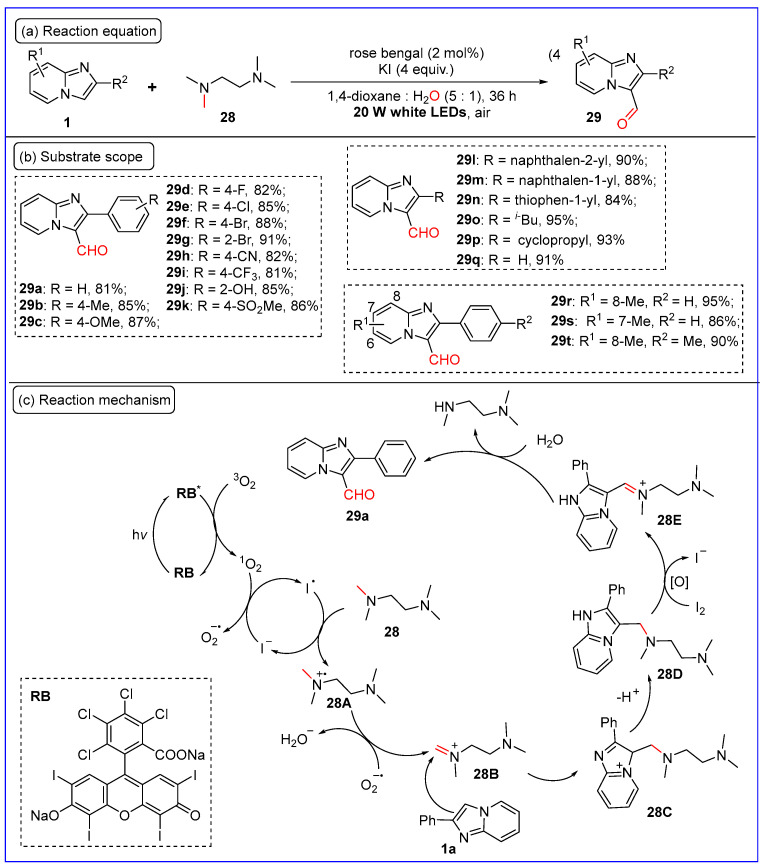
Visible light-induced C3-formylation of imidazo[1,2-a]pyridines with tetramethylethylenediamine (TMEDA). (**a**) Reaction equation. (**b**) Substrate scope of imidazo[1,2-a]pyridines. (**c**) The plausible reaction mechanism.

The formyl group is an important functional group in the derivatization of organic and pharmaceutical molecules, so the formylation of imidazopyridine has always been a research hotspot for chemists [52,53]. In 2018, Hajra’s group reported a visible light-induced rose bengal-catalyzed C3 formylation of imidazo[1,2a]pyridines with tetramethylethylenediamine (TMEDA) (Scheme 16a) [54]. As shown in Scheme 16b, this method exhibited good substrate applicability and provided the corresponding products in 81–95% yields. In addition, 2-phenyl imidazo[1,2a]pyridines (**1a**) and TMEDA were subjected to a gram-scale reaction (6 mmol), resulting in **29a** in a 79% yield (1.053 g). The control experiments indicated that the reaction may undergo the superoxide radical pathway. A plausible mechanism for the photocatalytic formylation process was proposed in Scheme 16c.

### 2.5. C3-Alkoxycarbonylation of Imidazo[1,2-a]pyridines

**Scheme 17:**
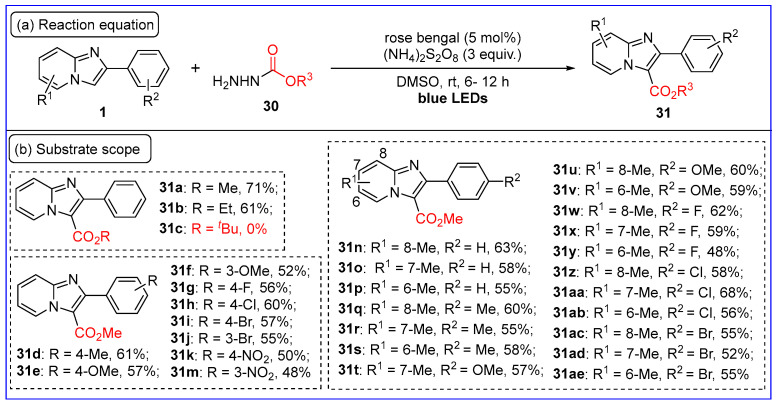
Visible light-induced C3-alkoxycarbonylation of imidazo[1,2-a]pyridines with carbazates. (**a**) Reaction equation. (**b**) Substrate scope.

Alkoxycarbonylation of imidazo[1,2-a]pyridine is a meaningful synthetic conversion, as the introduced ester groups [55] can be further converted into various functional groups, such as amide, hydroxymethyl, and carbonyl, etc. In 2022, Kshirsagar’s group developed a rose bengal-catalyzed C3-alkoxycarbonylation of imidazo[1,2-a]pyridines with carbazates by using (NH_4_)_2_S_2_O_8_ as a oxidant under blue LED illumination at room temperature (Scheme 17a) [56]. As shown in Scheme 17b, the substrate suitability study showed that this transformation has good functional group tolerance. Both methyl carbazate **30a** and ethyl carbazate **30b** were suitable for this reaction and provided the corresponding products **31a** and **31b** in 71% and 61% yields, respectively. However, tertbutyl carbazate could not smoothly convert to the desired product **31c** under the optimal conditions. The 2-arylimidazopyridines substrates with different substituents (such as methyl, methoxy, halogen, and nitro) were also compatible with this system, and afforded the corresponding products **31d**-**31ae** in moderate yields.

**Scheme 18:**
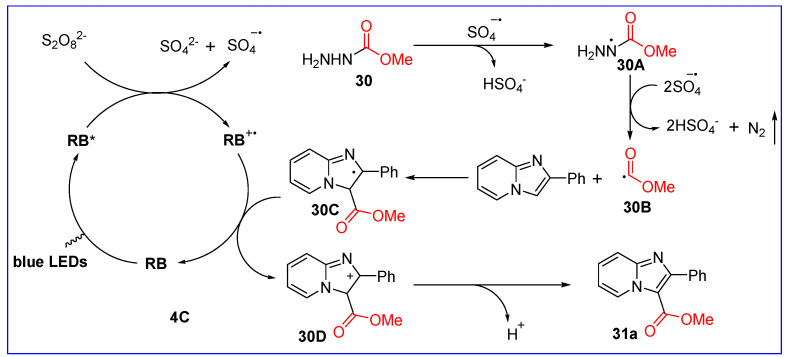
Proposed mechanism for alkoxycarbonylation of imidazo[1,2-a]pyridines.

Based on the results of control experiments, a plausible mechanism was proposed by the authors (Scheme 18). Firstly, the excited state species of rose bengal formed by visible-light irradiation reduced the persulfate to the sulfate radical anion via a single electron transfer. Subsequently, nitrogen radical intermediate **30A** was generated via hydrogen atom transfer between methyl carbamate and sulfate anions, which further underwent sequential dehydrogenation and released nitrogen molecules to form alkoxycarbonyl radical intermediate **30B**. Then, radical intermediate **30C** was formed through a free radical addition of **30B** and **1a**, which was further oxidized and deprotonated to obtain the final product **31a**.

### 2.6. C3-Arylation of Imidazo[1,2-a]pyridines

**Scheme 19:**
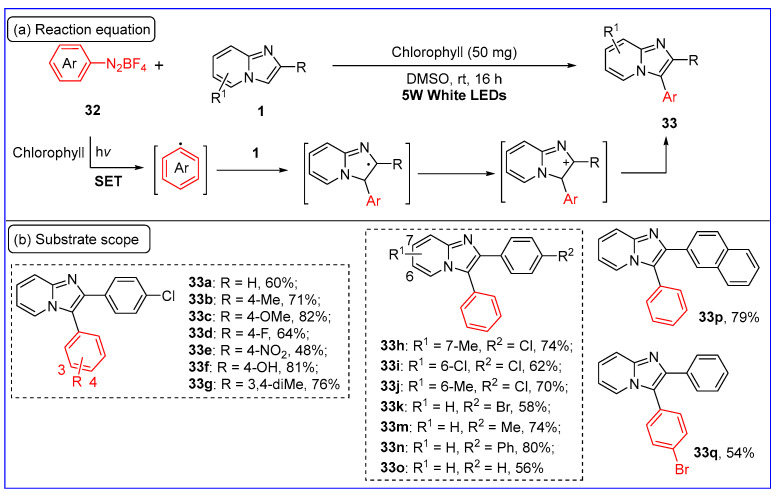
C3-arylation of imidazo[1,2-a]pyridines with diazonium salts. (**a**) Reaction equation. (**b**) Substrate scope.

Furthermore, 2,3-Diarylimidazo[1,2-a]pyridine derivatives have significant biological activity potential [57,58], so their synthesis methods have been highly studied. In traditional methods for the synthesis of 2,3-diarylimidazo[1,2-a]pyridine derivatives, the transition-metal-catalyzed C-H bond arylation of 2-arylimidazo[1,2-a]pyridines is the most common synthetic strategy, which inevitably leads to serious heavy metal pollution problems [59]. In 2022, Mahdavi’s group disclosed a visible light-mediated method for the arylation of imidazo[1,2 a]pyridines using diazonium salts **32** by using chlorophyll as a photocatalyst (Scheme 19a) [60]. Various imidazo[1,2-a]pyridines and diazonium salts bearing electron-withdrawing and electron-donating groups were well tolerated to construct 2,3-Diarylimidazo[1,2-a]pyridine compounds **33** in moderate to good yields (Scheme 19b).

### 2.7. C3-Carbosilylation of Imidazo[1,2-a]pyridines

**Scheme 20:**
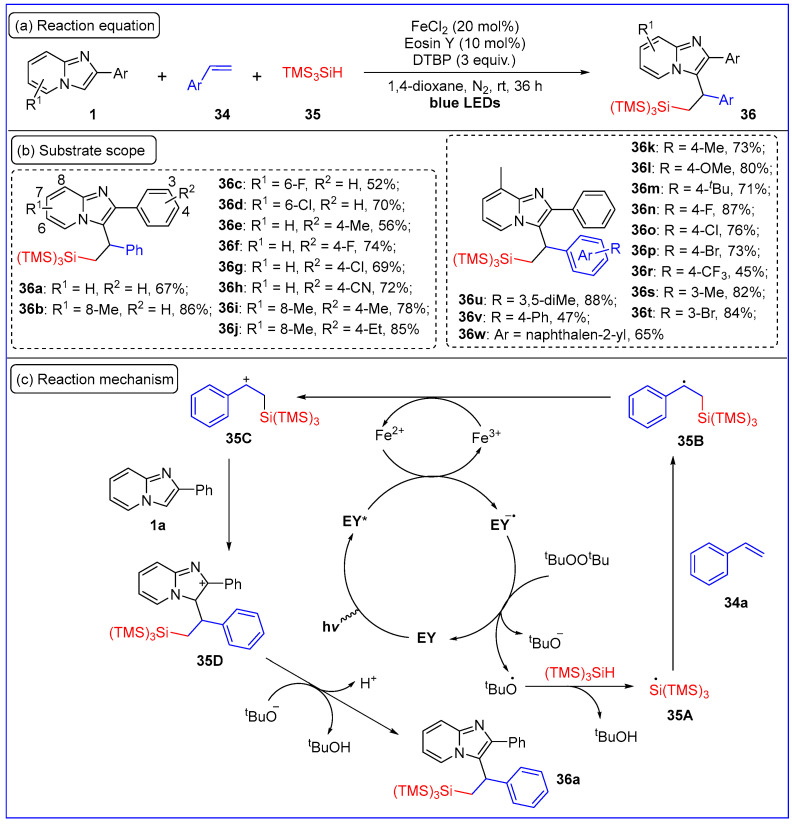
C3-carbosilylation of imidazo[1,2-a]pyridines with alkenes and silanes. (**a**) Reaction equation. (**b**) Substrate scope. (**c**) The plausible reaction mechanism.

The synthesis methodology of organosilicon molecules has attracted widespread attention from chemists due to these molecules’ significant applications in the fields of materials, polymer science, and agricultural chemistry. In 2021, Hajra’s group established a visible light-induced protocol for FeCl_2_ and eosin Y-catalyzed three-component carbosilylation of imidazo[1,2-a]pyridines with alkenes and silanes at room temperature (Scheme 20a) [61]. The substrates were studied systematically and obtained the corresponding products in satisfactory yields (Scheme 20b). This transformation exhibited good functional group tolerance and substrate adaptability.

A possible mechanism proposed by the authors was shown in Scheme 20c. Initially, radical anion EY^•−^ and Fe(III) were generated through a single electron transfer (SET) occurring from Fe(II) to the excited state of EY (EY*), which then transferred SET to DTBP to generate ^t^BuO• and regenerate the photocatalyst. (TMS)_3_Si• produced via the reaction of ^t^BuO• and silane (**35**) reacted with olefin (**34**) to form radical intermediate **35B**. Subsequently, **35B** underwent SET with iron(III) to obtain the regenerated Fe(II) catalyst and cationic intermediate **35C**. Then, the electrophilic addition process occurred between **35C** and imidazo[1,2-a]pyridine **1a** to obtain **35D**. Finally, **35D** underwent deprotonation to obtain the desired product **36**.

### 2.8. C5-Alkylation of Imidazo[1,2-a]pyridines

**Scheme 21:**
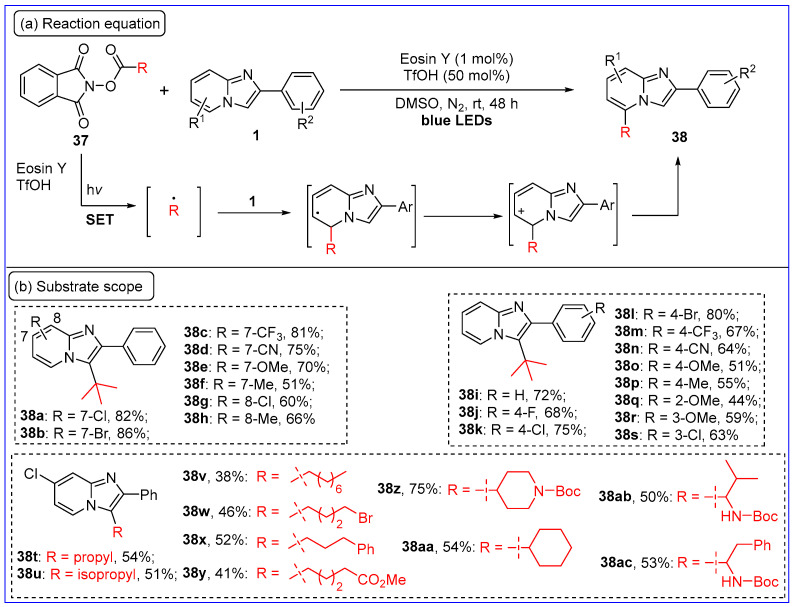
Visible light-induced C5-alkylation of imidazo[1,2-a]pyridines with alkyl *N*-hydroxyphthalimides. (**a**) Reaction equation. (**b**) Substrate scope.

At present, the C3 functionalization of imidazo[1,2-a]pyridine has been widely explored, but the visible light-induced C5 functionalization of imidazo[1,2-a]pyridines has still rarely been investigated [62]. In 2020, Jin’s group developed a visible light-induced method for the C5 alkylation of imidazo[1,2-a]pyridines with alkyl *N*-hydroxyphthalimides **37**, by using eosin Y as a photocatalyst at room temperature (Scheme 21) [63].

As shown in Scheme 21b, various imidazo[1,2-a]pyridine substrates containing different electron-donating groups (such as methyl and methoxy) and electron-withdrawing groups (such as fluorine, chlorine, bromine, trifluoromethyl, and cyano) on either C2-benzene or pyridine rings were all compatible with this reaction system, generating the corresponding products **38a**–**38s** in moderate to good yields. *N*-hydroxyphthalimides substrates **37** with different substituents (such as primary, secondary, and tertiary alkyl esters) were also suitable for this conversion, obtaining the corresponding products **38t**–**38ac** in 38–75% yields. In addition, gram-scale experiments have also been successfully completed under the standard conditions.

## 3. C-N Bond Formation

### 3.1. C3-Sulfonamidation of Imidazo[1,2-a]pyridines

**Scheme 22:**
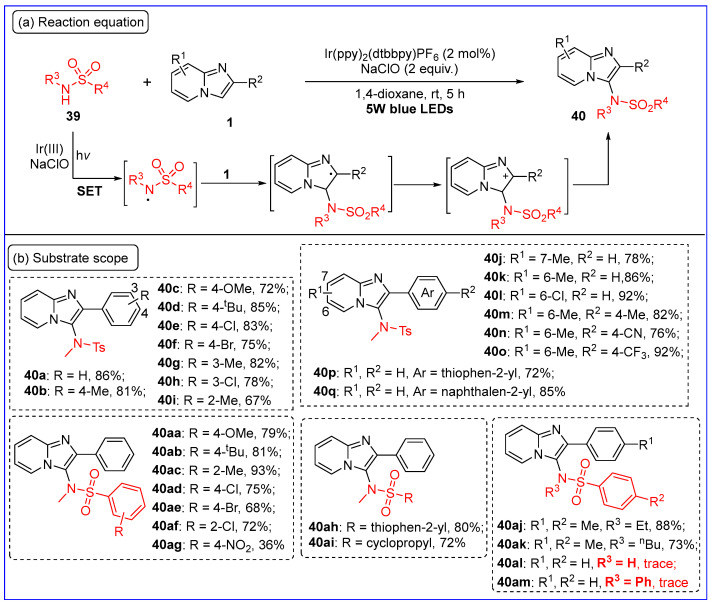
Visible light-induced C3-sulfonamidation of imidazo[1,2-a]pyridines with sulfamides. (**a**) Reaction equation. (**b**) Substrate scope.

Sulfonamide moieties are one of the most important pharmacophores in many clinical drugs, such as rosuvastatin and benazepril [64]. Therefore, the introduction of sulfonamide moieties on the *N*-heterocycle backbones is a hot topic of research for chemists [65]. In 2018, Sun’s group developed a visible light-promoted protocol for C3 sulfonamidation of imidazo[1,2-a]pyridines with sulfamides **39** by using NaClO solution as the oxidant and Ir(ppy)_2_(dtbbpy)PF_6_ as a photosensitizer under mild conditions (Scheme 22) [66].

Various imidazo[1,2-a]pyridine substrates with different electron-donating (such as Me, OMe, and ^t^Bu) and electron-withdrawing groups (such as Cl, Br, CN, andCF_3_, etc.) on either C2-aryl or pyridine rings were tolerated under the optimal reaction conditions and generated the corresponding products **40a**–**40q** in 67–92% yields (Scheme 22b). *N*-methyl-arylsulfonamide substrates containing different substituents could proceed smoothly and generated the desired products **40aa**–**40ai** in 36–93% yields. Furthermore, both *N*-ethyl-*p*-toluenesulfonamide and *N*-butyl-*p*-toluenesulfonamide were also suitable for this method, yielding the corresponding products **40aj** and **40ak** in 88% and 73% yields, respectively. Unfortunately, *N*-phenyl-benzenesulfonamide and benzenesulfonamide substrates failed to give the corresponding products **40al** and **40am**.

### 3.2. C3-Amination of Imidazo[1,2-a]pyridines

**Scheme 23:**
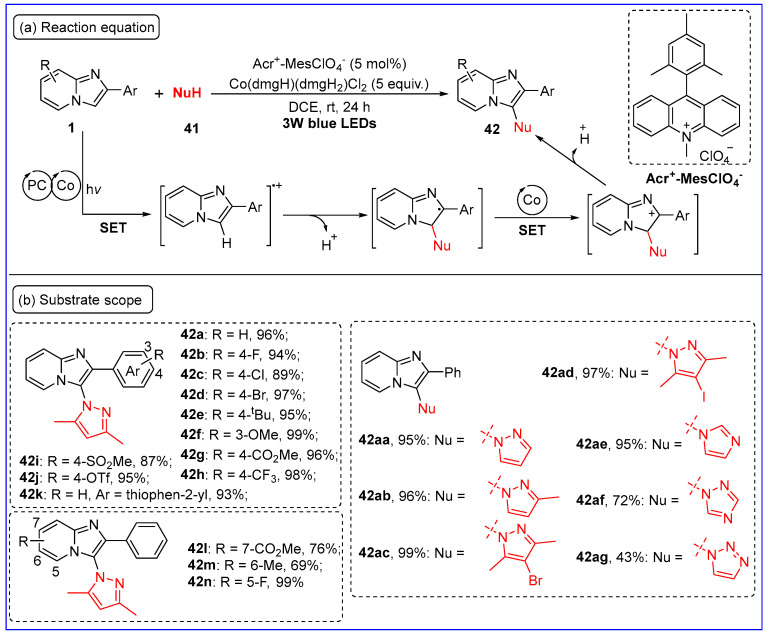
Visible light-induced C3-amination of imidazo[1,2-a]pyridines with azoles. (**a**) Reaction equation. (**b**) Substrate scope.

Heteroarylamines are prominent in many natural products, drugs, and functional materials [67]. Therefore, the direct C-H amination of heteroarenes has received widespread attention from synthetic and pharmaceutical chemists. In 2018, Lei’s group reported a photo-induced external oxidant-free protocol for C3-amination of imidazo[1,2-a]pyridines with azoles, by employing acridinium as a photosensitizer and cobaloxime as the catalyst at room temperature (Scheme 23) [68]. A series of 2-arylimidazo[1,2-a]pyridines substituted with various electron-donating and electron-withdrawing groups were well tolerated to obtain the corresponding products **42a**–**42n** in good to excellent yields (69–99%). Various types of azoles, including pyrazoles, imidazoles, 1,2,3-triazole, and 1,2,4-triazole, could also achieve this conversion to provide the corresponding products **42aa**–**42ag** in 43–99% yields. In addition, Adimurthy’s team also successfully achieved a similar amination reaction of imidazo[1,2-a]pyridines with heteroamines (such as benzotriazoles, benzoimidazoles, triazoles, pyrazoles, imidazoles, and indazoles) by employing Acr^+^-MesClO^4-^ as a photocatalyst and K_2_S_2_O_8_ as an oxidant [69].

## 4. C-P Bond Formation

**Scheme 24:**
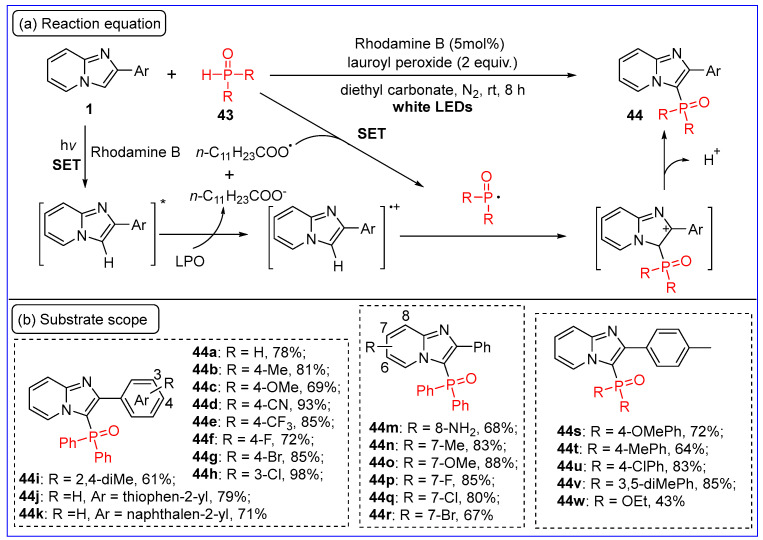
Visible light-induced C3-phosphorylation of imidazo[1,2-a]pyridines with phosphine oxides. (**a**) Reaction equation. (**b**) Substrate scope.

Phosphorus moieties are widely present in natural products, pharmaceutical molecules, and agricultural chemicals, possessing good physicochemical properties and certain bioactive activities [70]. The synthesis method of imidazo[1,2-a]pyridines containing phosphorus has also attracted the attention of chemists [71]. In 2021, Yu’s group reported a visible light-induced reaction for the phosphorylation of imidazo[1,2-a]pyridines with phosphine oxides **43** by using rhodamine B as a photocatalyst at room temperature (Scheme 24) [72]. A wide range of imidazo[1,2-a]pyridines and phosphine oxide substrates were systematically explored and provided the corresponding products **44a**–**44w** in 43–93% yields. The great advantages of this method are mild reaction conditions, wide substrate applicability, and good functional group tolerance.

## 5. C-O Bond Formation

**Scheme 25:**
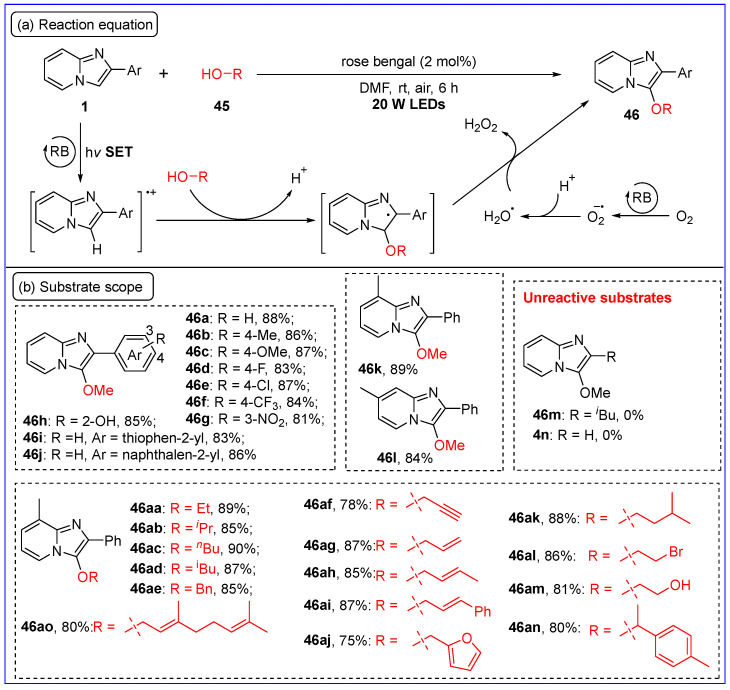
Photoredox-catalyzed C3-alkoxylation of imidazo[1,2-a]pyridines with alcohols. (**a**) Reaction equation. (**b**) Substrate scope.

Due to the widespread presence of C-O bonds in natural products and bioactive molecules, the development of efficient methods for constructing C-O bonds has received widespread attention from synthetic chemists [73]. In 2017, Hajra’s group achieved the visible light-induced C-3 alkoxylation of imidazopyridines with alcohols by using rose bengal as a photoredox catalyst under mild conditions (Scheme 25) [74]. The 2-arylimidazo[1,2-a]pyridines with various substituents (such as Me, OMe, F, Cl, CF_3_, NO_2_, and OH) on the phenyl ring could proceed smoothly and afforded the expected methoxylated products **46a**–**46h** in moderate to good yields. Both thiophen-2-yl and naphtha-2-yl-substituted imidazo[1,2-a]pyridines were well tolerated and gave the desired products **46i** and **46j** in 83% and 86% yields, respectively. However, no corresponding methoxylated products were detected under the standard conditions with C2-position-alkyl-substituted or -unsubstituted imidazo[1,2-a]pyridine substrates. Various primary and secondary alcohol substrates with alkyl, vinyl, alkynyl, hydroxyl, halogen, phenyl, and thiophene groups reacted well with 8-methyl-2-phenylimidazo[1,2-a]pyridine, providing the alkoxylation products **46aa**–**46an** in 75–90% yields.

## 6. C-S Bond Formation

### 6.1. C3-Thiocyanation of Imidazo[1,2-a]pyridines

Heterocyclic compounds containing thiocyanates are widely found in many natural products and have a wide range of biological activities [75]. Visible light mediated-thiocyanation is one of the effective methods for synthesizing organic heterocyclic thiocyanates [76].

**Scheme 26:**
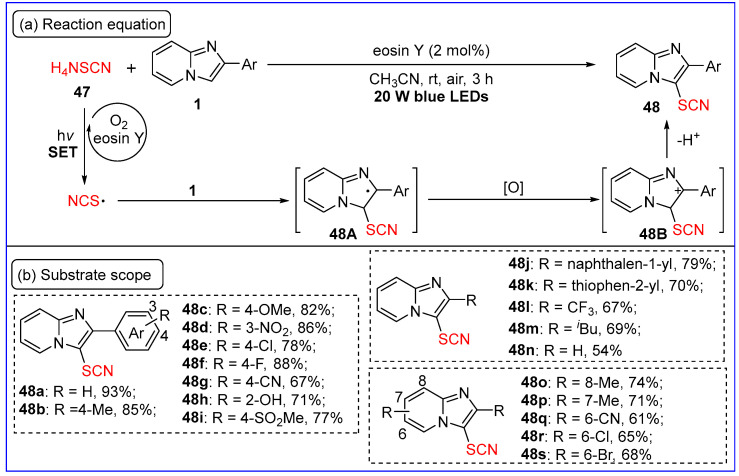
Photoredox-catalyzed thiocyanation of imidazo[1,2-a]pyridines with NH_4_SCN. (**a**) Reaction equation. (**b**) Substrate scope.

Hajra’s group developed a visible light-mediated protocol for the C3 thiocyanation of imidazo[1,2-a]pyridines with NH_4_SCN by using eosin Y as a photoredox catalyst in acetonitrile solution in an ambient air atmosphere (Scheme 26) [77]. A plausible mechanism of this reaction was investigated through the controlled experiments. Initially, thiocyanate radical was obtained from the oxidation of thiocyanate anion by SET mechanism via a reductive quenching cycle, which interacted with **1** to obtain the radical intermediate **48A**. Then, **48A** was oxidized to the cationic intermediate **48B**. Finally, **48B** underwent deprotonation to obtain the desired product **48** (Scheme 26a). For the substrate expansion experiment, 2-Aryl imidazo[1,2-a]pyridines with various electron-donating and electron-withdrawing groups on the phenyl ring were compatible with the reaction system to provide the corresponding products **48a**–**48i** in 67–93% yields. Trifluoromethyl and isobutylethyl-substituted or -unsubstituted substrates on the C2 position of imidazo[1,2-a]pyridines could also proceed smoothly under the optimal reaction conditions and obtained the corresponding products **48l**–**48n** in 54–69% yields. The gram-scale experiment of this conversion (6 mmol scale) was also performed and successfully afforded the target product in an 89% yield (1.34 g). In 2023, a similar photoredox-catalyzed methodology for the synthesis of 2-aryl-3-thiocyanatoimidazo-[1,2-a]pyridines by using naphthalimide as a photoredox catalyst was reported by Sharma’s group [78].

### 6.2. C3-Sulfonylation of Imidazo[1,2-a]pyridines

Heterocyclic compounds containing sulfone groups have a wide range of applications in the fields of agrochemicals, materials science, and medicinal chemistry. Therefore, the synthesis methodology for visible light-induced sulfonylation of imidazo[1,2-a]pyridines has been studied [79,80].

**Scheme 27:**
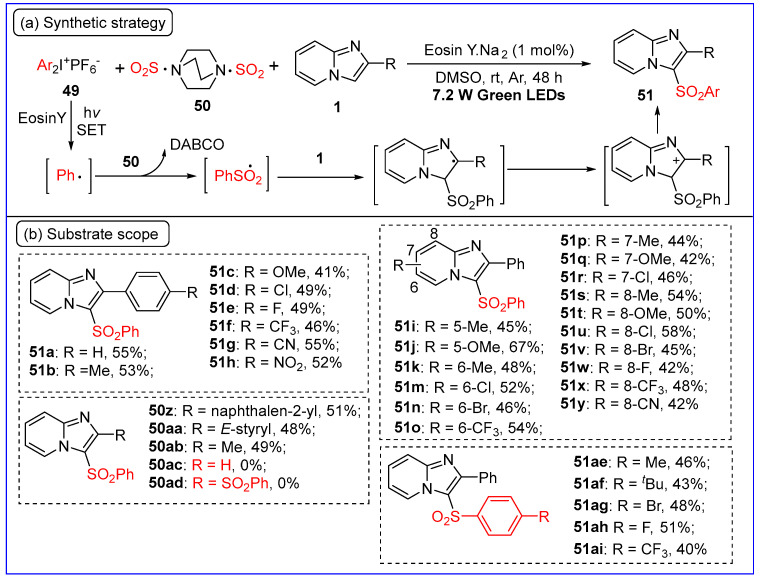
Photoredox-catalyzed C3-sulfonylation of imidazo[1,2-a]pyridines with diaryliodonium salts and DABCO-bis(sulfur dioxide). (**a**) Reaction straegy. (**b**) Substrate scope.

In 2020, Piguel’s group reported a visible light-induced three-component reaction for C3-sulfonylation of imidazo[1,2-a]pyridines **1** with diaryliodonium salts **49** and DABCO-bis(sulfur dioxide) **50** by using an organic photoredox catalyst at room temperature (Scheme 27) [81]. This method had good substrate compatibility and provided the corresponding products in moderate yields. However, C2 position-unsubstituted imidazo[1,2-a]yridine and 2-(phenylsulfonyl)imidazo[1,2-a]pyridine substrates are not suitable for this reaction system and failed to yield the sulfonylated products **50ac** and **50ad**, respectively.

### 6.3. C3-Sulfenylation of Imidazo[1,2-a]pyridines

**Scheme 28:**
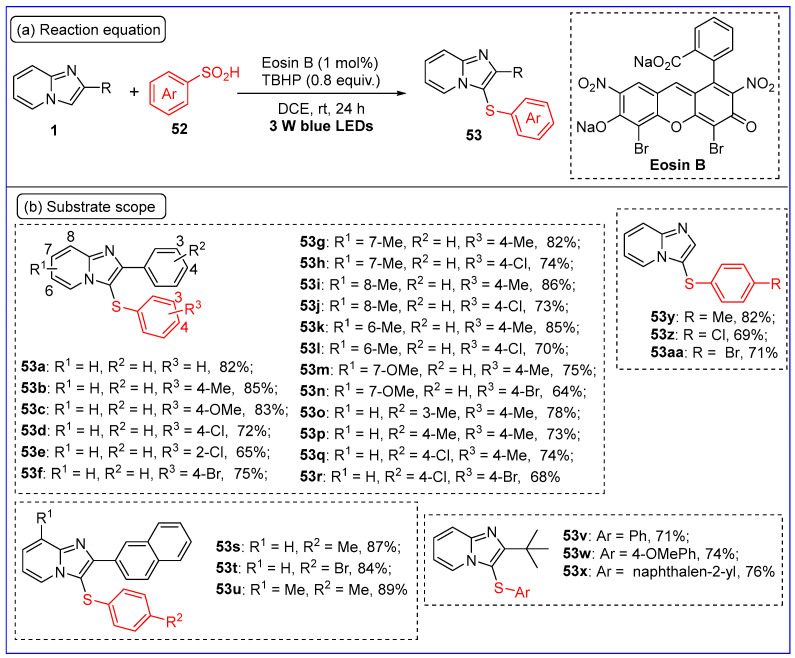
Photoredox-catalyzed C3-sulfenylation of imidazo[1,2-a]pyridines with sulfinic acids. (**a**) Reaction equation. (**b**) Substrate scope.

Considering that aryl sulfide moieties are widely present in bioactive molecules and natural products, visible light-induced direct sulfenylation of C-H bonds with sulfinic acids has been developed [82,83]. In 2017, Wang et al. developed a visible light-initiated method for the sulfenylation of imidazo[1,2-a]pyridines with sulfinic acids by using Eosin B as a photosensitizer and hydroperoxide (TBHP) as an oxidant in DCE at room temperature (Scheme 28a) [84]. For the substrate screening, a series of 2-arylimidazo[1,2-a]pyridines and sulfinic acid substrates bearing electron-donating groups as well as halogen substituents were well tolerated in this transformation, resulting in the corresponding imidazo[1,2-a]pyridine derivatives in moderate yields (Scheme 28b).

**Scheme 29:**
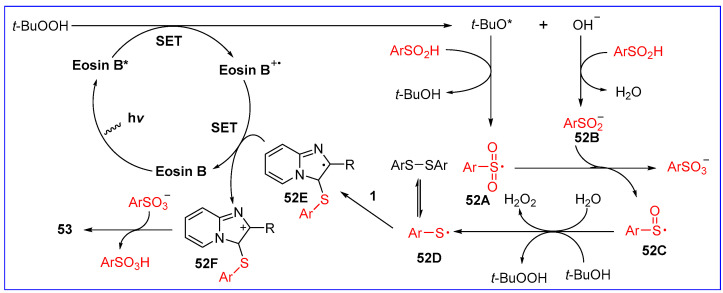
A proposed mechanism for the C3-sulfenylation of imidazo[1,2-a]pyridines.

A radical pathway mechanism was proposed based on the results of control experiments (Scheme 29). Initially, the excited Eosin B* generated from Eosin B via visible light irradiation underwent a single electron transfer (SET) process to TBHP, generating tertbutoxyl radical and a hydroxyl anion. Then, the arylsulfinic acid reacted with tertbutoxyl radical to obtain the sulfonyl radical **52A**. Subsequently, the sulfinyl radical **52C** was formed via the reaction of **52A** with arylsulfinate **52B**, which was reduced by ^t^BuOH or H_2_O to generate the thienyl radical intermediate **52D**. Then, **52D** underwent addition reaction with imidazo[1,2-a]pyridine **1** and further transformed into the carbocation intermediate **52F** through SET with Eosin B•+. Finally, **52F** underwent dehydrogenation under the action of the sulfonic acid anion and resulted in the desired product **53**.

**Scheme 30:**
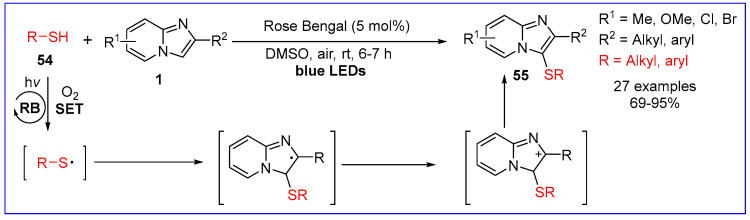
Photoredox-catalyzed C3-sulfenylation of imidazo[1,2-a]pyridines with thiols.

Soon afterwards, Barman’s group established another visible light-promoted procedure for the C3-sulfenylation of imidazo[1,2-a]pyridines with thiols by using rose bengal as a photoredox catalyst in ambient conditions (Scheme 30) [85]. This protocol could conveniently and efficiently provide a series of C3-sulfenylimidazo[1,2-a]pyridines in good to excellent yields, characterized by operational simplicity, broad substrate scope, and high atom efficiency.

## 7. C-Se Bond Formation

**Scheme 31:**
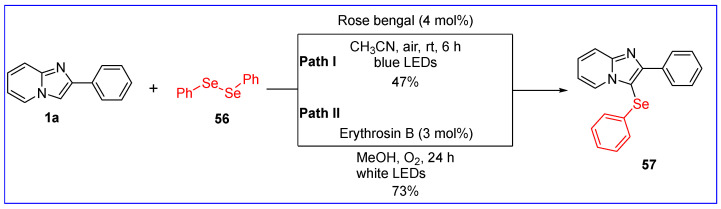
Photoredox-catalyzed C3-selenylation of imidazo[1,2-a]pyridines with diorganoyl diselenides.

Selenium is an important trace element that has multiple effects on human health, such as antioxidant effects, immune enhancement, liver protection, and anticancer effects [86]. Considering the increasingly important role of organic selenium in the field of medicinal chemistry, research on the selenylation of imidazo[1,2-a]pyridines has also attracted the attention of chemists [87]. For example, Braga’s group established a rose bengal-catalyzed protocol for the selenylation of imidazo[1,2-a]pyridines with diorganoyl diselenides in the presence of blue LEDs (415 nm) at room temperature, providing the 2-phenyl-3-(phenylselanyl)imidazo[1,2-a]pyridine **57** in a 47% yield (Scheme 31, Path I) [88]. In addition, Bagdi’s group also successfully developed a similar method for the selenylation of imidazo[1,2-a]pyridines by using erythrosine B as a photocatalyst. (Scheme 31, Path II) [89]. Notably, the yield of this method was better than that of Braga’s method.

## 8. Conclusions

In summary, significant progress has been made in recent years in the direct C-H functionalization of imidazo[1,2-a]pyridines based on arylation, thiolation, formylation, oxidative cycloaromatization, and carbonylation strategies, providing an effective pathway for the synthesis of various substituted imidazo[1,2-a]pyridine derivatives. This review mainly summarized the significant developments in the C3 position functionalization of imidazo[1,2-a]pyridines under visible light-induced conditions, and outlined the mechanisms and substrate applicability of various methods. It is worth noting that the visible light-induced C-H functionalization of imidazo[1,2-a]pyridines remains to be a hot and cutting-edge topic in organic synthesis methodology. At the same time, greener and more environmentally friendly methods conforming to the principle of green chemistry are still desirable to be developed. Therefore, we believe that these methods can provide excellent supplements for the photochemical construction of heterocyclic compounds. This not only provides assistance for the C-H functionalization of other heterocyclic compounds, but also provides new research inspiration for the visible light-catalyzed C-H functionalization of imidazo[1,2-a]pyridines in the future.

Obviously, research on the C-H functionalization of imidazo[1,2-a]pyridines mainly focuses on its C3 position. To date, reports are rarely available on the functionalization of other positions of imidazo[1,2-a]pyridines. We believe that through the continuous efforts of synthetic chemists, the study of C-H functionalization of imidazo[1,2-a]pyridines in other positions promoted by visible light will lead to breakthroughs, and will contribute to the development of organic synthesis and drug synthesis.
